# Supplementary material for: Learning curve and surgical outcome of robotic assisted colorectal surgery with ERAS program
Source: Sci Rep. 2022 Nov 29;12:20566. doi: 10.1038/s41598-022-24665-w (PMC9709162; doi:10.1038/s41598-022-24665-w)
Supplement: Supplementary file 1 — Supplementary Information 1. [file 41598_2022_24665_MOESM1_ESM.zip › LSC Raw data-Ñ[▒K/081/H_ñw╝╨░O▒Kñσ.pdf]

正式摘要(電子簽章：00000193698496)		
W52 / W52 ☐ 英文標題(注意:無英文姓名-請補病人基本檔!)	☐ 【讀取】憑證	

臺中榮民總醫院

出院病歷摘要

轉入號 : 轉出號 :

[redacted]

(8)流水編號	
(9)入院日期	109/06/18 (2020) 直腸外科 W52 - 162
(10)轉科日期	
(11)出院日期	109/06/30 (2020) 住院天數計 12 日

診斷

- 入院 : 1. Adenocarcinoma of A colon, s/p biopsy 20200604
  2. HCC, icteric type, s/p ERBD 20200513
  3. HBV carrier
  4. HTN
- 出院 : 1. Adenocarcinoma of A colon, s/p biopsy 20200604, s/p R't hemicolectomy on 20200619
  2. HCC, icteric type, s/p ERBD 20200513, s/p liver biopsy on 20200619
  3. HBV carrier
  4. HTN

主訴

- scheduled for operation for tissue proof and treatment

病史

- This is a 72 y/o man with history of HTN and HBV carrier, who presented with jaundice at first at Tzu-Chi. H and Klatskin tumor or HCC was impressed with IHD dilatation, s/p ERBD and HCC was impressed upon S4 and S6 also upon image. During admission period at Tzu-Chi. H, colonoscope was done and Adenocarcinoma of A colon was diagnosed. This time, he is admitted for operation of laparoscopic R't hemicolectomy and liver biopsy.

體檢發現

- Anal inspection:
  Recto digital examination:
  Sigmoidoscopy:
  Anoscopy:
  Abdomen:
  - General appearance :
    - easy looking , conscious clear , without cardiopulmonary distress
    - Vital signs自動帶入date 體溫(BT) 脈搏(P) 呼吸(R) 血壓(BP) 身高(BH) 體重(BW) BMI
      20200618 36.7℃ 93次/分 19次/分 137/87mmHg 137cm 87kg 46.4
  - HEENT :
    - Eyes: no anemic conjunctiva, no icteric sclera, normal EOM
    - ENT: no otorrhea, no rhinorrhea, no erythematous mucosa, no injected tonsil, no deformity
  - Neck: supple, freely movable, trachea midline,no deformity, no engorged jugular vein , no palpable thyroid or lymph node enlargement
  - Chest:
    - Inspection: Grossly normal, symmetric and fully expansion, no accessory muscle used, no operation scar, no spider angiomata, no gynecomastia
    - Palpation: no palpable mass or nodule, no subcutaneous emphysema, no tenderness
    - Ausculation: clear breathing sound
  - Heart:

-
    - Percussion: normal heart size
    - Ausculation: regular heart beat, normal S1, S2, no gallop, no murmur
- Abdomen:
    - Inspection: in
        - flat contour, symetrically, no scar, no stria, no dilated veins, no rashes
    - Ausculation: normoactive bowel sound, no abdominal bruits
    - palpation: soft, no muscle guarding or rigidity, no local tenderness, no rebounding pain, no palpable mass, no palpable pulsation
    - Percussion: no shifting dulllness, no hepatomegaly, no splenomagaly
-

Peripheral pulsation :	CA	BA	RA	FA	PA	PTA	DPA
Right side :	++	++	++	++	++	++	++/+++
Left side :	++	++	++	++	++	++	++/+++

- Genito-anus: no tenderness or palpable mass lesion or rectal shelf, normal external genitalia, normal anal tone, brown stool

CRS CONDITION

-
    - Lung

手術日期及方法

- 手術日期及方法20200619 Laparoscopy Liver biopsy
  20200619 Laparoscopic right colectomy and anastomosis

住院治療經過

- After admission, operation was done on 6/19. Post operative ileus happened and NG decompression was done which was self limited after then. Drainage was removed on 6/23. Try liquid diet on 6/27 and try semiliquid diet on 6/29. Due to relative stable condition, MBD was permitted and OPD f/u is scheduled.

合併症

一般檢查

特殊檢查

病理報告

其它

出院時情況

- 3.改門診治療

出院指示

藥名	劑量	劑量單位	用法	頻次	數量	數量單位	現況	天數	單位
Gaslan tab 40mg	1	TAB	PO	BID	14	TAB	TH	7	天
Urief tab 4 mg	1	TAB	PO	BID	14	TAB	TH	7	天
TramTOR cap 50mg	1	CAP	PO	Q6HPRN	28	CAP	TH	7	天

出院計劃

主治醫師 2062F 蔣鋒帆 / 住院醫師 2310K 陳璿夫

正式摘要(電子簽章：**000000193698496**)

W52 / W52

doctorRank:B
